# Strategic offloading of delayed intentions into the external environment

**DOI:** 10.1080/17470218.2014.972963

**Published:** 2014-11-18

**Authors:** Sam J. Gilbert

**Affiliations:** ^a^ Institute of Cognitive Neuroscience, University College London, London, UK

**Keywords:** Distributed cognition, Intentions, Prospective memory, Internet, Metacognition, Reminders

## Abstract

In everyday life, we often use external artefacts such as diaries to help us remember intended behaviours. In addition, we commonly manipulate our environment, for example by placing reminders in noticeable places. Yet strategic offloading of intentions to the external environment is not typically permitted in laboratory tasks examining memory for delayed intentions. What factors influence our use of such strategies, and what behavioural consequences do they have? This article describes four online experiments (*N* = 1196) examining a novel web-based task in which participants hold intentions for brief periods, with the option to strategically externalize these intentions by creating a reminder. This task significantly predicted participants' fulfilment of a naturalistic intention embedded within their everyday activities up to one week later (with greater predictive ability than more traditional prospective memory tasks, albeit with weak effect size). Setting external reminders improved performance, and it was more prevalent in older adults. Furthermore, participants set reminders adaptively, based on (a) memory load, and (b) the likelihood of distraction. These results suggest the importance of metacognitive processes in triggering intention offloading, which can increase the probability that intentions are eventually fulfilled.

Competent behaviour often requires us to form intentions for future actions, which cannot be fulfilled immediately. Several experimental paradigms have been developed to investigate this ability. Some of them fall within the domain of “prospective memory” (PM; Brandimonte, Einstein, & McDaniel, 1996; Kliegel, McDaniel, & Einstein, 2008), an umbrella term denoting a variety of processes that allow us to fulfil delayed intentions, at a variety of timescales (Craik & Kerr, 1996; Ellis & Cohen, 2008). PM paradigms typically require participants to perform an ongoing task while trying to remember to perform an intended action when they encounter a particular cue or at a particular time. Other conceptually related paradigms have been described as investigating “multitasking” (Burgess, Veitch, de Lacy Costello, & Shallice, 2000; Roca et al., 2011), “cognitive branching” or memory for “pending” intentions (Koechlin, Basso, Pietrini, Panzer, & Grafman, 1999), “memory for goals” (Altmann & Trafton, 2002), “goal neglect” (Bhandari & Duncan, 2014; Duncan, Emslie, Williams, Johnson, & Freer, 1996; Duncan et al., 2008), and “sustained attention” (Robertson, Manly, Andrade, Baddeley, & Yiend, 1997). These different terms have been used in related but nonidentical ways, and the boundary conditions separating the various paradigms are not always clear. Thus, in the present article, the theoretically neutral term “memory for delayed intentions” is used to refer to the multiple processes supporting the execution of intended behaviours that can only be fulfilled after performance of an interposed activity.

One characteristic of the experimental paradigms referenced above is that they rarely, if ever, give participants the opportunity to create external reminders. However, in everyday life we often augment our memory for delayed intentions with external artefacts (Hall, Johansson, & de Léon, 2013; Harris, 1980). We tie knots in handkerchiefs, make notes in to-do lists and calendars, physically hold task-relevant objects (e.g., a letter that needs to be posted), place objects in noticeable places, or ask friends and partners to remind us. Today, people increasingly programme time-, location-, or person-based reminders into smartphones (Svoboda, Rowe, & Murphy, 2012). In other words, we often “outsource” or “offload” our intentions into the external environment, at least to some degree, rather than relying on a purely internal representation. In this way, our intentions are represented in a system that extends beyond our brains and bodies into distributed physical artefacts and our social worlds. In order to understand how we fulfil delayed intentions, it is therefore important to consider this distributed system.

The phenomenon of distributed cognition is recognized within many fields of psychology (Clark, 1997, 2010; Hutchins, 1995; Kirsh, 1995). It is also explored in philosophy (Clark & Chalmers, 2006; Menary, 2010), with implications and practical applications across many domains such as user-interface design (Wright, Fields, & Harrison, 2000). An obvious example is the use of pen and paper to record information, which can subsequently be consulted rather than relying on unaided memory. As well as using the external environment as a repository of representational information, we also physically interact with the world to reduce the computational load of subsequent information processing. For example, expert Tetris players tend to physically rotate pieces as they fall down the screen to check their perceptual match with unfilled spaces, rather than relying on a slow and unreliable mental rotation strategy. Popular behaviour management techniques such as “getting things done” (Allen, 2002) emphasize the importance of offloading intended tasks into an external memory (Heylighen & Vidal, 2008). In these ways, we restructure our environment to create perceptual triggers for appropriate behaviour (Kirsh, 1996), rather than relying on more computationally demanding cognitive processes.

Theoretical models of how we remember delayed intentions emphasize the flexible balance between perceptual triggering (e.g., being reminded to post a letter by the sight of a mailbox) versus strategic monitoring (e.g., continually searching for a mailbox; Gilbert, Hadjipavlou, & Raoelison, 2013; McDaniel & Einstein, 2000; Scullin, McDaniel, & Shelton, 2013). While some studies have investigated the efficacy of experimenter-provided reminders (Guynn, McDaniel, & Einstein, 1998; Henry, Rendell, Phillips, Dunlop, & Kliegel, 2012; Loft, Smith, & Bhaskara, 2011; Vortac, Edwards, & Manning, 1995), the literature on delayed intentions has rarely examined the ways that we manipulate the environment ourselves to create perceptual triggers. One exception to this has been observational studies of the way that people remember intentions in workplace settings such as nursing or aviation (Grundgeiger, Sanderson, MacDougall, & Venkatesh, 2010; Loukopolous, Dismukes, & Barshi, 2009). Another exception is the clinical literature on rehabilitation (Fish, Wilson, & Manly, 2010; Thöne-Otto & Walther, 2008; Wilson, Emslie, Quirk, & Evans, 2001). However, in many ways, the problems facing patients are similar to those faced by neurologically healthy adults, and failures to fulfil delayed intentions are common even in highly able individuals. In the words of Duncan (2010, p.93), “the frontal lobe patient is very much like the rest of us—but more so”. Yet experimental studies of neurologically healthy participants have not generally permitted the creation of external cues (though see Einstein & McDaniel, 1990, for one exception).

The present study therefore had two aims. The first was to develop a simple task permitting the use of an externalizing strategy, to investigate (a) whether participants voluntarily offload intentions, even when they can use unaided memory alone if they prefer, and (b) whether intention offloading is influenced by task characteristics, suggesting an influence of metacognitive insight into the likelihood of forgetting. Two characteristics were manipulated: (a) the memory load (i.e., number of concurrent intentions to be remembered), and (b) the presence of an interruption in the ongoing task (Altmann, Trafton, & Hambrick, 2013). Both factors were hypothesized to increase the difficulty of remembering intentions and thus to prompt increased intention offloading. While the experimental task developed here involved intentions that were delayed only for a few seconds, the second aim of the present study was to combine this task with a real-world intention operating over several days, to test its external validity in predicting everyday behaviour extended over a longer timescale.

## EXPERIMENT 1A

### Method

#### Intention-offloading task

**Figure 1:**
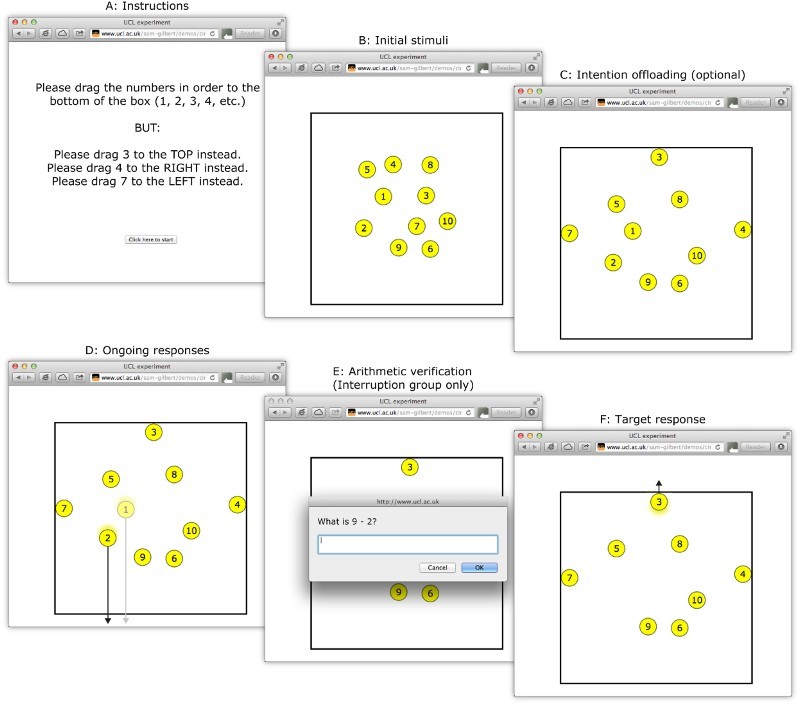
Schematic illustration of the intention-offloading task.

Participants completed the task via their computer's web browser. On each trial, 10 yellow circles numbered 1–10 were positioned randomly within a box (Figure 1). Participants were instructed to drag the circles in turn (1, 2, 3, etc.) to the bottom of the box, using their computer mouse. When each circle was dragged to the bottom of the box it disappeared, leaving the other circles on the screen. After the 10th circle had disappeared, the screen was cleared, and the next trial began (for a demonstration, please visit http://www.ucl.ac.uk/sam-gilbert/demos/circleDemo.html).

Alongside this ongoing task, participants were provided with delayed intentions on each trial. They were instructed to drag one circle (1-target condition) or three circles (3-target condition) to specific alternative locations (i.e., left, right, or top). Thus, participants formed delayed intentions to perform particular actions when they encountered prespecified cues, although they could produce a standard ongoing response (i.e., dragging the circle to the bottom of the box) if they forgot. However, if they attempted to drag a target circle to an incorrect target location (left, top, or right), or if they attempted to drag a nontarget circle to any of these locations, it remained on the screen, allowing participants to realize that they had made a mistake.

This task permits intention offloading in a simple manner: At the beginning of each trial, participants could drag the target circles towards their intended location. From this point on, there is no need to mentally rehearse the delayed intention(s). Instead, the locations of the target circles themselves represent the intention, providing a perceptual trigger when they are reached in the sequence. An everyday analogue might be leaving an object by the front door, so that we remember to take it with us when leaving the house. Participants were explicitly told that they could use this strategy if they wished, but they were also told it was optional and it was up to them whether to use it. Half of the participants performed the task as described (“no-interruption group”). The other participants (“interruption group”) additionally received a distracting arithmetic question during each trial. This occurred immediately after dragging one of the nontarget circles to the bottom of the box, at a position in the sequence randomly selected between the first circle and the circle immediately before the first target. Each participant completed 20 experimental trials, 10 in the 1-target condition and 10 in the 3-target condition, in randomized order. Participants were instructed to perform the task as quickly and accurately as possible.

#### Participants

Participants were recruited from the Amazon Mechanical Turk website (http://www.mturk.com), an online marketplace in which participants receive payment for completion of web-based tasks (Crump, McDonnell, & Gureckis, 2013). Ethical approval was received from the UCL (University College London) Research Ethics Committee, and informed consent was obtained from all participants. Participation was restricted to volunteers living in the USA, to reduce heterogeneity. A total of 100 participants were recruited; two were excluded due to poor arithmetic-verification performance (<80%) and were replaced with a further two participants (final sample: mean age 33 years, range 18–62 years, 43% male). The experiment took approximately 20 min, and participants were compensated $2.

#### Data analysis

There were two dependent measures. *Target accuracy* was the proportion of targets that were dragged to their instructed location rather than the bottom of the screen. Only trials in which the target was dragged to the instructed location on the first attempt were counted as correct (i.e., if a participant first tried to drag a circle to the wrong location, found that it remained on the screen, then tried again at the correct location, this was not counted as correct). *Externalizing proportion* was the proportion of targets for which participants set up an external reminder, by moving it to a new location before reaching its position in the ongoing task (see Supplemental Material for full details of how this was calculated).

### Results

**Figure 2:**
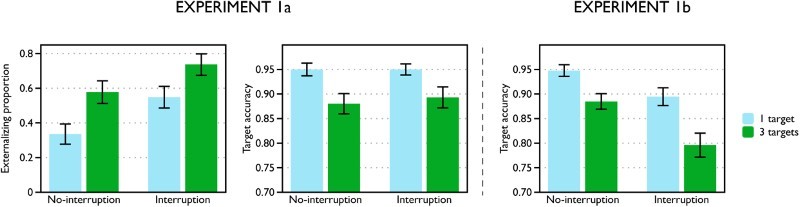
Mean externalizing proportion and target accuracy in Experiments 1a and 1b. Error bars indicate standard error of the mean.

Mean arithmetic-verification accuracy was 99%. The mean retention intervals (i.e., time from the start of each trial until the first target circle was reached in the sequence) were 13.8 s and 9.0 s in the interruption and no-interruption groups, respectively. Other results are summarized in Figure 2. The mean externalizing proportion was significantly greater than zero in all conditions, *t*(49) > 5.7, *p* < .001, *d* > 1.6, indicating that participants did set reminders at least on a proportion of trials. The externalizing proportion was significantly greater for 3-target than 1-target trials, *F*(1, 98) = 43, *p* < .001, η^2^ = .31, and for the interruption than the no-interruption group, *F*(1, 98) = 5.2, *p* = .025, η^2^ = .051. These two factors did not significantly interact (*F* < 1).

Analysis of target accuracy showed that participants dragged the target circles to their instructed locations on a high proportion of trials (>88% in all conditions). Accuracy was higher for 1-target than 3-target trials, indicating that participants were more likely to miss targets when they had a higher memory load, *F*(1, 98) = 23.6, *p* = .000004, η^2^ = .194. There was no significant difference in accuracy between the interruption and no-interruption groups, nor did the Group × Memory Load factors significantly interact, *F*(1, 98) < 0.3, *p* > .6, η^2^ < .003.

**Figure 3:**
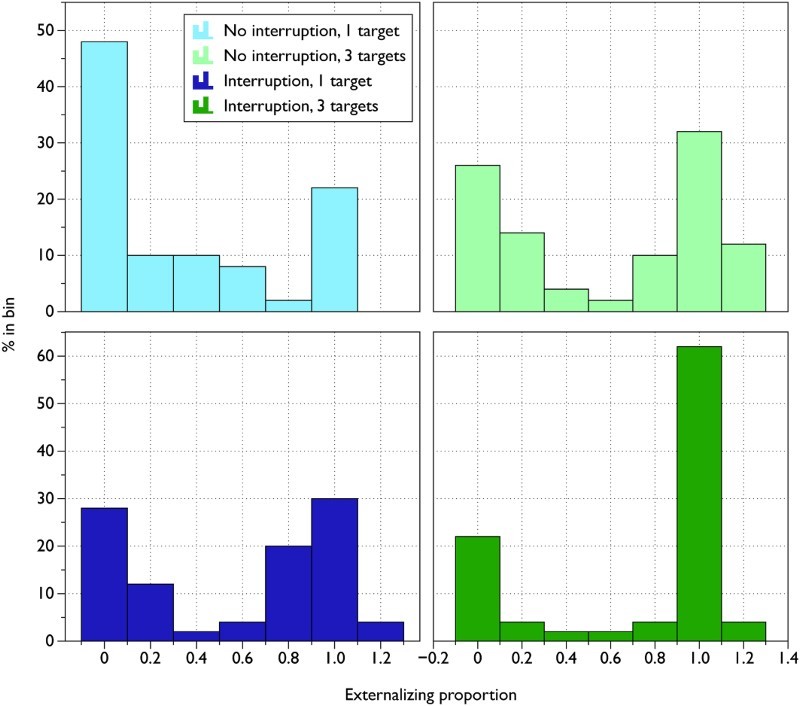
Frequency histograms of the externalizing proportion in each condition. To view this figure in colour, please visit the online version of this Journal.

In order to investigate whether intention offloading may have functionally contributed to target accuracy, the correlation between each participant's externalizing proportion and target accuracy was calculated. A significant positive correlation was observed in both the no-interruption (*r* = .29; *p* = .04) and the interruption (*r* = .46; *p* = .0008) groups. In all four conditions, the distribution of externalizing proportions was bimodal rather than normally distributed (Kolmogorov–Smirnoff test: all *p*s < .00002; see Figure 3). Thus, individual participants tended to either always or never externalize, rather than externalizing on an intermediate proportion of trials. Note that an externalizing proportion greater than 1 is possible if participants move a target circle more than once, before reaching its position in the sequence.

### Discussion

Participants voluntarily created external reminders, and they did so flexibly based on (a) the mnemonic demands of the task, and (b) the characteristics of the ongoing task in which delayed intentions were embedded. Furthermore, individuals who set more reminders fulfilled their delayed intentions more often. Individual participants tended to either always externalize or never do so. This suggests that there is relatively little advantage in testing participants for a large number of trials; instead a design in which a large number of participants each complete a small number of trials is most efficient.

Participants' adaptive use of an externalizing strategy suggests that their metacognitive awareness of the likelihood of forgetting (i.e., more likely when there is a higher memory load, or a more distracting ongoing task) prompted compensatory offloading of intentions (see Maylor, 1990, for a related finding). However, this interpretation rests on the untested assumption that interruption would have impaired participants' ability to remember delayed intentions if they had been unable to offload them. The following experiment tests this assumption.

## EXPERIMENT 1B

### Method

The same task was administered, except that only the upcoming circle in the sequence could ever be dragged; the other circles remained fixed in place (i.e., Circle 2 could only be moved after Circle 1 had disappeared; Circle 3 could only be moved after Circle 2 had disappeared, etc.). This made it impossible to offload intentions, and thus intention-offloading instructions were not presented. Methods were otherwise identical to those in Experiment 1a. In this experiment, and all subsequent experiments reported below, participants who had already taken part in an earlier experiment were blocked. This was achieved by blocking the Amazon Mechanical Turk ID code of any participant who had taken part in an earlier study, as well as any IP (Internet protocol) address that had previously been used to access one of the studies.

#### Participants

A total of 100 participants were recruited, divided equally between the interruption and no-interruption groups. One participant was replaced due to arithmetic-verification accuracy below 80% (final sample: mean age 31 years, range 18–59 years, 43% male).

### Results

Mean arithmetic-verification accuracy was 99%. The mean retention intervals (which, in this experiment, did not include time spent creating external reminders) were 11.7 s and 6.8 s in the interruption and no-interruption groups, respectively. See Figure 2 for target accuracy. As in Experiment 1a, accuracy was higher for 1-target than 3-target trials, *F*(1, 98) = 38.5, *p* < 10^−7^, η^2^ = .28. However, unlike the previous experiment, accuracy was now lower in the interruption than in the no-interruption group, *F*(1, 98) = 10.4, *p* = .002, η^2^ = .096. The two factors did not significantly interact, *F*(1, 98) = 1.9, *p* = .17, η^2^ = .019. A cross-experiment comparison showed that the predicted Experiment × Interruption interaction was significant, *F*(1, 196) = 6.7, *p* = .011, η^2^ = .033. The experiment factor did not interact significantly with any other factor, *F*(1, 196) < 1.8, *p* > .18, η^2^ < .09.

### Discussion

Interruption clearly impaired the fulfilment of delayed intentions when participants relied on their unaided memory. But when they could offload intentions in Experiment 1a, it led to greater offloading without affecting accuracy. This suggests that in some circumstances participants can eliminate the costs of interruption by offloading intentions to the external environment. Thus, intention offloading can play a compensatory role, presumably influenced by metacognitive insight into the conditions likely to disrupt performance.

Having shown that participants adaptively set external reminders in a laboratory task, an obvious question remains. What reason is there to believe that the present intention offloading/control tasks relate to the real-world behaviours of interest when we try to remember delayed intentions in everyday life? It might be hoped that laboratory investigations of tasks requiring participants to execute delayed intentions will relate to important real-world behaviours such as remembering to take medication, to attend appointments, and so on. However, it is not clear that the present experimental tasks have any real-world significance, unless they can be shown to relate in some way to a theoretically relevant naturalistic behaviour. Experiment 2 seeks to address this question by investigating the relationship between the present experimental paradigm and a naturalistic intention embedded into participants' everyday lives over a period of days, rather than seconds. A further aim of Experiment 2 was to test the possibility that the intention-offloading task administered in Experiment 1a might correlate better with naturalistic PM than the nonoffloading control task administered in Experiment 1b. This is because only the former task allows participants to set external reminders, like many real-world situations, but in contrast with standard laboratory tasks.

## EXPERIMENT 2

In the introduction to this article, a variety of methodological approaches were described for experimentally assessing participants' ability to remember delayed intentions. One of the most striking differences between these paradigms is the retention interval—that is, the time between encoding an intention and the opportunity to act on it. Whereas the tasks administered in Experiment 1 had a short retention interval, on the order of 5 to 15 s on average, real-world intentions operate over a wide variety of durations, ranging from a few seconds (e.g., momentarily delaying a pending task during periods of high workload in an aviation setting) to periods of minutes, hours, days, or longer (e.g., remembering to attend a planned hospital appointment). It is therefore an open question how much overlap there is between processes that allow us to fulfil “immediate intentions”, delayed by just a few seconds, versus delayed intentions operating over longer periods.

In the literature on prospective memory, a distinction has been made between “vigilance” tasks, potentially involving conscious rehearsal of a delayed intention over a short time scale, versus “PM proper”, in which participants do not continuously rehearse their delayed intention but instead must bring it back to consciousness at the appropriate time (Graf & Uttl, 2001). The present intention-offloading task would be more akin to a vigilance task than PM-proper, according to this terminology. While delayed intention tasks operating over different timescales will undoubtedly rely on at least partially distinct mechanisms, it is not clear to what extent they also overlap. For example, rostral prefrontal cortex appears to play a prominent role in prospective memory in everyday life (Burgess, 2000; Uretzky & Gilboa, 2010) as well as remembering intentions for just a few seconds (Gilbert, 2011), whereas more posterior frontal regions respond more strongly to standard working memory tasks (Reynolds, West, & Braver, 2009). This suggests that there may be some overlap between the processes that allow the realization of intentions over a few seconds and those operating over longer timescales; however, the inferential basis for this type of “reverse inference”, arguing for shared cognitive process on the basis of similar neurophysiological response, is relatively weak (Poldrack, 2006, 2011). Thus, the following experiment investigates behaviourally whether the present experimental paradigms can be related to participants' fulfilment of naturalistic intentions operating over longer periods of time and embedded within everyday activities. In order to do this, participants' performance of the tasks administered in Experiment 1 was measured, along with a more naturalistic measure of their real-world ability to fulfil delayed intentions over a longer period. Performance of more traditional (a) event- and (b) time-based PM tasks was also investigated, requiring participants to act on a delayed intention (a) when a particular cue occurred, or (b) at a particular time. Participants additionally performed a lexical decision task within which these two PM tasks were embedded, yielding a more general measure of cognitive ability and task engagement (note that Ratcliff, Thapar, & McKoon, 2010, Table 4, found a correlation of .72 between lexical decision accuracy and Wechsler Adult Intelligence Scale–Third Edition, WAIS–III, IQ).

### Method: Experiment 2a

#### Tasks

##### Intention-offloading/Nonoffloading task

Participants in the offloading condition performed the same task as that in Experiment 1a (permitting intention offloading), whereas participants in the nonoffloading condition performed the same task as that in Experiment 1b (disallowing intention offloading). In both cases, only the no-interruption version was administered. As in Experiment 1, participants performed 10 trials with one target and 10 trials with three targets.

##### Lexical decision task

Participants were presented with a sequence of upper-case letter strings (3–5 letters). They responded with their right middle finger to words (50% of trials) and their right index finger to nonwords. After each response the stimulus was removed, and the next trial followed after 150 ms. Participants were instructed to perform the task as quickly and accurately as possible.

##### Event-based PM task

Participants performed the lexical decision task described above, with the additional instruction that if they saw an animal word (e.g., COW) they should press a button with their left index finger instead. A total of 265 trials were performed, including 10 targets.

##### Time-based PM task

Participants again performed the lexical decision task. They were also asked to press a button with their left index finger every 30 s. They were told that they needed to make this response within 3 s of the correct time for it to count. Participants could press a button with their left middle finger at any point to reveal a digital clock at the top of the screen. This indicated the time since the beginning of the task, staying on the screen for 1500 ms. The task lasted for 310 s, yielding 10 opportunities to make a PM response.

##### Naturalistic PM task.

Participants were informed that they had the opportunity to earn three bonus payments of $0.25. They were provided with a unique weblink and were instructed that they would earn $0.25 if they visited this link on three named dates that were 2, 5, and 7 days after the test. Participants completed the experiment on a Wednesday, so the dates for the bonuses were the following Friday, Monday, and Wednesday. They were also asked at this point whether they intended to claim each of the three bonuses. Only participants reporting an intention to claim all three bonuses were included in data analysis.

#### Procedure

After providing consent, participants performed the tasks in counterbalanced order, with the constraint that the two lexical decision tasks were performed successively. Prior to these two tasks, participants first performed 20 practice trials of the lexical decision task alone. Instructions for the time- and event-based PM tasks were provided immediately prior to task performance, and for the second of these tasks participants were informed that the previous PM instructions no longer applied. The instructions for the naturalistic PM task were presented at the end of the session, after performance of the other tasks. Participation took approximately 20–30 minutes, for which participants received $2.

#### Participants

A total of 675 participants were recruited (mean age: 32 years, range 18–67 years; 49% male; 337 in the offloading condition and 338 in the nonoffloading condition). After applying planned exclusion criteria to ensure adequate performance of all tasks and an intention to claim all three bonuses (see Supplemental Material), 439 participants were retained for analysis (217 in the offloading condition and 222 in the nonoffloading condition; mean age: 31 years, range 18–62 years; 51% male).

### Method: Experiment 2b

One concern with the intention offloading/nonoffloading task administered in Experiment 2a is that target accuracy is generally high, resulting in little variance in performance. In order to reduce possible ceiling effects, an additional study was conducted in which participants performed a more difficult version of the task. There were two changes. First, participants were required to perform an interleaved task between receiving the instructions for each trial and beginning to drag circles to the bottom of the screen. A set of eight blue circles appeared at the bottom of the screen, each containing a letter. Participants were required to rearrange these circles so that they spelled the word “CONTINUE” from left to right, upon which they disappeared, and the task could be continued. This led to a much longer retention interval; however, intention offloading in the offloading group was permitted before this interposed task was attempted. The second discrepancy with Experiment 2a was that the numbered circles were not always yellow but were filled with a variety of colours (black, brown, blue, red, green, yellow, orange, white, pink, and grey). Target circles were specified in terms of their colour rather than their number (e.g., please drag the blue circle to the left). The allocation of colours to numbers was randomly chosen on each trial. This change was based on the distinction in the PM literature between “focal” and “nonfocal” tasks. Focal tasks are those in which the stimulus characteristic defining the target(s) overlaps with a characteristic that must also be attended in order to perform the ongoing task (e.g., in Experiment 2a the numbers that define the target circles must also be attended in order to drag the nontarget circles in sequence to the bottom of the screen). By contrast, the target-defining characteristic in Experiment 2b was not relevant to the ongoing task. Note that nonfocal PM tasks have been proposed to rely on top-down monitoring to a greater degree than focal tasks (Scullin, McDaniel, & Einstein, 2010). Thus the aim of this experimental manipulation was not to reduce the extent to which the intention offloading task required target monitoring—that is, “vigilance” as opposed to “PM-proper”. Rather, the aim of this manipulation was to depress performance levels so that there might be greater variance between participants which might be related to the other measures. Apart from these adjustments to the intention-offloading/nonoffloading task, the procedure in Experiment 2b was identical to that in Experiment 2a.

#### Participants

A total of 826 participants were recruited (mean age: 32 years, range 18–68 years; 46% male; 449 randomly allocated to the offloading condition, 377 to the nonoffloading condition). After applying identical exclusion criteria to Experiment 3a, 557 participants were retained for analysis (303 in the offloading condition and 254 in the nonoffloading condition; mean age: 32 years, range 18–67 years; 48% male).

### Results

Task performance is summarized in Table 1. Performance on all measures was similar between Experiments 2a and 2b (comparison between experiments: all *p*s > .19), except for the intention-offloading/nonoffloading task, which in Experiment 2b was performed with lower accuracy, *F*(1, 992) = 63, *p* < 10^−12^, η^2^ = .06, and with a greater externalizing proportion in the offloading group, *F*(1, 518) = 111, *p* < 10^−22^, η^2^ = .18. Collapsing across the two experiments and the two groups (offloading and nonoffloading), 47% of participants failed to claim any bonuses, despite reporting that they intended to claim all three, 11% claimed one bonus, 13% claimed two bonuses, and 28% claimed three. These results are consistent with previous studies indicating large discrepancies between participants' self-reported intentions and their subsequent behaviour in real-world tasks (Sheeran, 2002). Furthermore, the distribution of scores shows that participants were most likely to claim all of the bonuses or none of them, rather than an intermediate number. The number of bonuses claimed did not differ between participants in the two experiments and two groups, χ^2^(9) = 13.7, *p* = .13. Lexical decision reaction times (RTs) were longer in the event-based than in the time-based condition, *F*(1, 994) = 544, *p* < 10^−95^, η^2^ = .35, potentially as a result of additional processing of each word in the event-based task to check its target status, before making a response.

**Table 1.** Performance measures in Experiments 2a and 2b

	**Experiment 2a**				**Experiment 2b**			
	Nonoffloading		Offloading		Nonoffloading		Offloading	
	M	SD	M	SD	M	SD	M	SD
*Event-based PM task*								
Lexical decision (RT/ms)	768	114	764	114	780	137	777	127
Lexical decision (% correct)	95.3	2.6	95.1	2.5	95.1	2.7	95.1	2.8
PM (% hits)	67.8	20.2	67.4	20.0	68.5	19.7	67.1	19.8
*Time-based PM task*								
Lexical decision (RT/ms)	713	111	718	114	721	115	723	118
Lexical decision (% correct)	95.1	2.9	94.9	3.4	94.9	3.3	95.3	2.8
PM (% hits)	82.7	21.8	83.5	20.7	79.0	24.4	81.9	22.5
*Intention-offloading/nonoffloading task*								
1-target condition (% hits)	93.7	9.5	94.7	7.8	84.1	16.1	92.6	10.3
3-target condition (% hits)	88.2	11.5	90.6	9.3	79.8	18.3	89.1	11.5
1-target condition (externalizing proportion)	—	—	.33	.42	—	—	.75	.40
3-target condition (externalizing proportion)	—	—	.67	.45	—	—	.90	.30
Naturalistic PM (% bonuses claimed)	38.9	41.5	41.0	43.8	42.5	44.3	40.9	43.9

*Note:* PM = prospective memory; RT = reaction time.

In Experiment 2a the mean retention intervals were 9.7 s and 7.3 s in the offloading and nonoffloading groups, respectively; the equivalent figures in Experiment 2b were 24.4 s and 26.0 s. In the offloading condition, the externalizing proportion was significantly greater for 3-target than for 1-target trials, as in Experiment 1a [Experiment 2a: *F*(1, 216) = 158; *p* < 10^−26^; η^2^ = .42; Experiment 2b: *F*(1, 302) = 73; *p* < 10^−15^; η^2^ = .20]. In both experiments, target accuracy was significantly higher in the offloading than in the nonoffloading groups [Experiment 2a: *F*(1, 437) = 4.8; *p* = .029; η^2^ = .011; Experiment 2b: *F*(1, 555) = 71.7, *p* < 10^−15^; η^2^ = .11], indicating that intention offloading functionally contributed to performance.

Correlations between performance measures are shown in Tables 2 and 3. Table 2 shows results collapsed over all 996 participants, which is most appropriate for evaluating the naturalistic PM, event-based PM, time-based PM, and lexical decision accuracy measures, seeing as these were identical for all participants (furthermore, ongoing lexical decision accuracy was collapsed over the event-based and time-based task). The intention-offloading/nonoffloading task is also included for reference, collapsed over the 1-target and 3-target trials for all participants. Table 3 shows the results from these measures separately for the two experiments and two groups of participants (offloading and nonoffloading).

**Table 2.** Correlations between measures collected in Experiments 2a and 2b

	Naturalistic PM	Event-based PM	Time-based PM	Intention-offloading/nonoffloading	Lexical decision accuracy
Naturalistic PM	—	.07*	.06^#^	.13***	.07*
Event-based PM		—	.02	.18***	.43***
Time-based PM			—	.14***	.10**
Intention-offloading/nonoffloading				—	.25***
Lexical decision accuracy					

*Note:* PM = prospective memory.

^#^ *p* < .1. *p < .05. **p < .01. ***p < .001.

**Table 3.** Correlations between measures collected in Experiments 2a and 2b, separately for the two experiments and two groups

	Naturalistic PM	Event-based PM	Time-based PM	Intention-offloading/nonoffloading	Lexical decision accuracy
*Experiment 2a*					
Naturalistic PM	—	.05	.12#	.09	.19**
Event-based PM	.02	—	.11	.24***	.44***
Time-based PM	.10	.03	—	.22**	.18**
Intention-offloading/nonoffloading	.15*	.25***	.19**	—	.41***
Lexical decision accuracy	.08	.41***	.14*	.23***	—
Externalising proportion	−.02	−.02	.00	.11	−.01
*Experiment 2b*					
Naturalistic PM	—	.04	.01	.22***	.01
Event-based PM	.15**	—	.04	.26***	.45***
Time-based PM	.04	−.05	—	.06	.09
Intention-offloading/nonoffloading	.10^#^	.12*	.13*	—	.26***
Lexical decision accuracy	.04	.43**	.02	.19***	—
Externalizing proportion	−.02	.08	−.07	.32***	.09

*Note:* PM = prospective memory. Shaded cells: intention-offloading group; white cells: nonoffloading group.

^#^ *p* < .1. *p < .05. **p < .01. ***p < .001.

Considering Table 2 to begin with, the correlation coefficients between the accuracy measures were universally positive, as commonly found in such analyses of psychometric tests (Spearman, 1904). However, perhaps surprisingly, the time- and event-based PM tasks were not significantly correlated, suggesting that they are supported by distinct cognitive mechanisms. Both tasks were significantly correlated with the intention-offloading/nonoffloading task, indicating their reliability as measures. They were also significantly correlated with lexical decision accuracy, particularly the event-based task, potentially due to the shared reliance of the ongoing and target-detection demands on lexical processing. Correlations between the naturalistic PM task and other measures were modest but nevertheless significant for the event-based PM task (*p* = .025) and lexical decision task (*p* = .027), marginally significant for the time-based PM task (*p* = .069), and highly significant for the intention-offloading/nonoffloading task (*p* = .00004).

Turning now to the subsamples shown in Table 3, the only significant correlation between the naturalistic PM task and the other accuracy measures, after Bonferroni correction for 16 tests (four measures × four groups) was with the nonoffloading task of Experiment 2b (corrected *p* = .005). At an uncorrected threshold, the naturalistic PM task had at least a marginally significant correlation with the intention-offloading/nonoffloading task in three of the four groups. The other tasks each had at least a marginally significant correlation with the naturalistic PM task in one of the four groups.

It thus appears that the validity of the experimental tasks for predicting naturalistic PM performance was low, but somewhat higher for the intention-offloading/nonoffloading task than for the other measures. In order to further examine this possibility, a multiple regression was conducted, attempting to predict the number of bonuses claimed in the naturalistic PM task from (a) event-based PM accuracy; (b) time-based PM accuracy; (c) lexical decision accuracy; (d) intention-offloading/nonoffloading task; (e) Intention-Offloading/Nonoffloading × Experiment interaction; (f) Intention-Offloading/Nonoffloading × Group interaction; (g) Intention-Offloading/Nonoffloading × Experiment × Group interaction. Of these seven predictors, only one was significant: intention-offloading/nonoffloading task performance [standardized beta = .13, *t*(988) = 3.8, *p* = .00017; all other predictors: *t*(988) < 1.8, *p* > .07]. This result was not due to collinearity between the event-based PM, time-based PM, and lexical decision measures. When the same analysis was run three times, comparing the intention-offloading/nonoffloading task with the other tasks one by one, the intention-offloading/nonoffloading predictor was always highly significant, *t*(990) > 4.0, *p* < .00005, but none of the other three was, *t*(990) < 1.35, *p* > .17. Thus, the intention-offloading/nonoffloading task outperformed each of the three other measures for explaining variance in naturalistic PM, even when these measures were considered individually. Furthermore, bootstrap tests (Grömping, 2006) to directly compare the relative importance of the intention-offloading/nonoffloading task against the other three measures (using a default of 1000 bootstrap runs) yielded an effect at *p* < .1 in each case.

**Figure 4:**
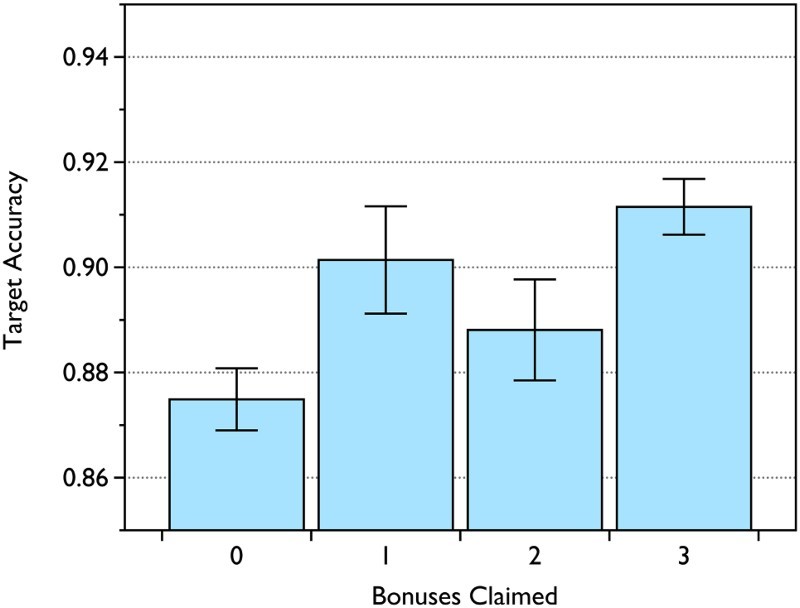
Target accuracy in the intention-offloading/control task against the number of bonuses claimed in the naturalistic PM (prospective memory) task. Error bars indicate standard error of the mean.

In order to test whether the predictive validity of the intention-offloading/nonoffloading task was jointly significant in the offloading and nonoffloading groups, the regression analysis was repeated separately for each group, after dropping predictors (f) and (g). In both independent samples, the intention-offloading/nonoffloading task uniquely predicted the number of bonuses claimed [offloading: *t*(514) = 2.1, *p* = .038; nonoffloading: *t*(470) = 3.5, *p* = .0005; all other tasks: *p* > .09]. In the nonoffloading group, there was also a significant effect of the regressor representing the Nonoffloading Task × Experiment interaction, *t*(470) = 2.3, *p* = .024, indicating that the predictive validity of the measure was greater in the more difficult version of the task. However, in the offloading group the predictive validity of the intention-offloading task did not differ between the easy and the difficult versions of the task, *t*(514) = 0.20, *p* = .84. The relationship between number of bonuses claimed in the naturalistic PM task and mean performance in the intention-offloading/nonoffloading task is shown in Figure 4.

These results might reflect, trivially, the fact that there were more targets in the intention-offloading/nonoffloading task (20 trials, half of which had three targets) than in the event- and time-based PM tasks (10 targets each), perhaps leading to additional statistical power for this measure. In order to test this possibility, the multiple regressions were repeated using only 1-target performance as a predictor rather than the collapsed score, so that the intention-offloading/nonoffloading task was matched to the PM tasks in having only 10 targets. Results remained similar: The intention-offloading/nonoffloading task uniquely predicted the number of bonuses claimed when combining across the two groups, *t*(988) = 3.2, *p* = .001, and also in both independent samples [offloading: *t*(514) = 2.3, *p* = .02; nonoffloading: *t*(470) = 2.6, *p* = .01; all other tasks: *p* > .09].

Another concern is that mean performance of the intention-offloading/nonoffloading task was relatively high, leading to possible ceiling effects. However, if anything this would reduce its ability to capture variance related to the naturalistic PM task, whereas in fact it was a better predictor than the event- and time-based PM tasks, which were performed with lower accuracy. The number of bonuses claimed formed a non-normal (bimodal) distribution, violating one of the assumptions for significance testing of Pearson correlations. While it has been suggested that the Pearson correlation is “extremely robust” and can withstand violations of assumptions such as normality (Field, 2000, p. 87), it is nevertheless necessary to examine whether this could have compromised significance testing of the correlation between the naturalistic PM task and other measures, particularly the intention-offloading/nonoffloading task, which showed the highest correlation. To test this possibility, the naturalistic PM variable was randomly shuffled, and its correlation with the intention-offloading/nonoffloading target accuracy was calculated. This procedure was repeated 1,000,000 times for each of the four conditions shown in Table 3, as well as the collapsed data shown in Table 2. In every case, 5.0% of these tests produced a significant result at *p* < .05, apart from Experiment 2a, nonoffloading group, where 4.9% of tests were significant. Thus, Type 1 errors were appropriately controlled.

One of the aims of Experiment 2 was to investigate whether allowing participants to set external reminders might increase the external validity of the intention-offloading task, relative to the nonoffloading control task. In fact, there was no significant difference between the two versions of the task, and, if anything, the association with the naturalistic PM measure was numerically greater in the nonoffloading task. However, it should be noted that allowing participants to offload intentions boosted their performance, thus reducing variance that could be linked with naturalistic PM performance. In order to examine the potential influence of this factor, an additional analysis was undertaken, in which the offloading and nonoffloading groups were matched in performance. This was achieved by repeatedly removing the best scoring participant in the offloading group and the worst-scoring participant in the nonoffloading group, separately for each experiment, until the two groups were as closely matched as possible. As a result, 10 participants in each group (5%) were removed from Experiment 2a, and 58 (21%) from Experiment 2b (seeing as there was a greater difference between the two groups in Experiment 2b, this required the removal of more participants). The resulting mean target accuracies were 92.27%/92.25% in the offloading/nonoffloading groups of Experiment 2a, and 88.72%/88.83% in Experiment 2b. Correlations between the intention-offloading/nonoffloading task and the naturalistic PM measure were then recalculated, yielding the following results: Experiment 2a offloading: *r* = .15, *p* = .03, nonoffloading: *r* = .09, *p* = .19; Experiment 2b offloading: *r* = .11, *p* = .08, nonoffloading: *r* = .06, *p* = .43. Thus, after matching groups for mean target accuracy, the intention-offloading task significantly predicted naturalistic PM in Experiment 2a, and did so marginally significantly in Experiment 2b, but there was no significant correlation with the nonoffloading task in either experiment. Collapsing across both experiments, the predictive validity of the intention-offloading task was highly significant (*p* = .009) but the predictive validity of the nonoffloading task was not significant (*p* = .36). Nevertheless, the difference between these associations was not itself statistically reliable (*p* = .25).

### Discussion

Two features of the naturalistic PM task are particularly striking. First, participants performed quite poorly: Even though all analysed participants reported an intention to claim all three bonuses, about half failed to claim a single one. Second, the number of bonuses collected was only weakly related to performance of the experimental tasks. There are a number of reasons why this might be: Some participants may have lost their note of the weblink used to claim their bonuses, or copied it down incorrectly; they may have lacked motivation to claim small bonuses of only $0.25; they may have had technical problems preventing them from accessing the specified weblink (e.g., internet connectivity problems); they may have found themselves unexpectedly busy or away from their computers on the specified days (up to a week away), and so on. The naturalistic PM score is therefore likely to be an exceedingly noisy measure, a problem exacerbated by the collection of only three “trials”.

In this context, it is perhaps also striking that predictions of naturalistic PM from the intention-offloading/nonoffloading task were nevertheless highly statistically significant (*p* < .00008), albeit reflecting a weak effect size (η^2^ = .022). This relationship is unlikely to merely reflect general motivation/engagement with the experimental tasks. The lexical decision measure was pooled from hundreds of trials and yet the 1-target intention-offloading/nonoffloading measure, drawn from just 10 trials and with many participants performing at ceiling, was the best predictor of naturalistic PM in two independent samples (note that although lexical decision accuracy was generally high, no participant scored a perfect 100%, indicating that ceiling effects were avoided). This suggests that the intention-offloading/nonoffloading task is of interest as a paradigm that captures variance related to a theoretically interesting real-world behaviour. Indeed, the intention-offloading/nonoffloading task uniquely predicted variance in this behaviour when controlling for all other measures.

In the time- and event-based PM tasks, participants were informed of the delayed intentions immediately prior to task performance. Although performance of these tasks was somewhat below ceiling, suggesting that participants did not continuously rehearse their delayed intentions throughout these tasks, it is unclear whether other PM tasks involving longer, distraction-filled, retention intervals might be more likely to predict naturalistic PM. A study by Uttl and Kibreab (2011) found that naturalistic PM was not predicted by such a task, while the best predictor of naturalistic PM in the present study was a task with a relatively brief retention interval. Nevertheless, this is an interesting question for future research.

Even when analysis of the intention-offloading/nonoffloading task was restricted to the 10 trials in the 1-target condition, this task was a better predictor of naturalistic PM than the event- and time-based PM tasks. One possible explanation of this might be that it involved encoding of 10 different intentions, one for each trial, as opposed to 10 opportunities to fulfil a single intention as in the event- and time-based tasks. This could have led to a better sampling of processes related to encoding new intentions, rather than simply maintaining and acting on a single established intention. Related to this possibility, the use of a single intention in the event- and time-based tasks could have led to a progressive automatization of the PM response in these tasks, reducing the validity of the more traditional PM tasks for predicting the fulfilment of a novel intention. By contrast, the intention-offloading/nonoffloading task may have required participants to manage interference between currently relevant and previously relevant intentions, which may more accurately reflect everyday PM. In order to engage such processes in the more traditional PM tasks, it would be necessary to switch between multiple intentions rather than acting on a single intention throughout.

It is debatable whether the intention-offloading/nonoffloading task should be considered an example of a PM task or not. Certainly, this task requires participants to retain intentions over a shorter timescale than standard PM paradigms, and it seems more likely to involve continuous rehearsal of an intention over the retention period, rather than bringing the intention to mind at the appropriate moment. It might be more appropriately considered a working memory (WM) rather than a PM task. However, the conceptual advance engendered by this label is unclear, seeing as WM is itself an umbrella term encompassing diverse experimental paradigms, from maintenance of perceptual, spatial or verbal information to complex “n-back” and “span” tasks, with uncertain construct validity (Kane, Conway, Miura, & Colflesh, 2007; Shallice & Cooper, 2011). Rather than debating this terminological question, it is perhaps more fruitful merely to note two points. (a) The intention-offloading/nonoffloading task undoubtedly requires participants to act on a delayed intention, in the sense that they are provided with an instruction on each trial that they can only fulfil after a (brief) filled delay; and (b) it is an open question to what extent the processes contributing to this task overlap with those studied in traditional PM paradigms operating over longer time periods. Nevertheless, the intention-offloading/nonoffloading task was the best predictor of participants' fulfilment of a theoretically interesting behaviour—that is, acting on a naturalistic intention delayed for up to one week, embedded within everyday activities. This provides an empirical demonstration of its relevance to the process of remembering delayed intentions in everyday life. These points stand regardless of whether the intention-offloading/nonoffloading task is itself classified as a PM task, a WM task, or indeed some other sort of task. Further studies, perhaps involving a larger battery of tests, might help to delineate in more detail the properties that confer external validity for predicting naturalistic PM onto an experimental task.

### Investigation of age effects

Previous studies of PM have noted a divergence between laboratory-based and real-world tasks. For instance, although age-related deficits in laboratory tasks have consistently been reported (Uttl, 2008), there is little evidence for an equivalent deficit in naturalistic tasks, with some studies even reporting age-related improvement (Phillips, Henry, & Martin, 2008). This is the “age-related PM paradox”. Some authors have suggested that this divergence may be accounted for, at least in part, by more frequent or effective use of external reminders in older participants as a compensatory mechanism (see Maylor, 2008; Phillips et al., 2008, for discussion). Moreover, ageing has been hypothesized to lead to an increasing reliance on environmental support across a range of tasks, not just those involving PM (Lindenberger & Mayr, 2014). Thus, although it is not the main focus of the present studies, it is potentially interesting to investigate whether older participants made greater use of external reminders in the intention-offloading task. Of the 1196 participants contributing to the analyses reported above, the median age was 29 years with a range from 18–67 years; 214 participants (18%) were 40 or above, 91 (8%) were 50 or above, and 18 (2%) were 60 or above. Previous analyses have suggested that age differences in PM are small until participants are in their 50s or 60s (Uttl, 2008), but detectible at a younger age if the sample is large enough (Logie & Maylor, 2009).

In the present studies, correlation coefficients between age and externalizing proportion were generally positive but nonsignificant (Experiment 1a: *r* = .12, *p* = .22; Experiment 2a: *r* = .13, *p* = .05; Experiment 2b: *r* = .11, *p* = .07). In order to pool these results into a single analysis, a multiple regression was conducted to predict each participant's externalizing proportion from their age, along with dummy variables coding for each of the separate experiments. This revealed a significant positive relationship between age and externalizing proportion, *t*(616) = 3.0, *p* = .003. A further analysis attempting to predict target accuracy in the intention-offloading task did not show an effect of age, *t*(616) = 0.06, *p* = .95. However, for participants performing the nonoffloading task there was a significant positive relationship with age, *t*(572) = 1.99, *p* = .047. In order to follow up this finding, an additional regression analysis was conducted in which all participants were included, with regressors representing age, group (offloading, nonoffloading), and Age × Group interaction, and dummy variables representing the different experiments. This showed a positive effect of age, *t*(1190) = 1.97, *p* = .049, and also a significant effect of group, *t*(1190) = 3.8, *p* < .0002 (i.e., performance was better in the offloading group). However, the Age × Group interaction was not significant, *t*(1190) = 1.3, *p* = .21. Thus, there was no significant difference between the age effects in the offloading versus nonoffloading groups, and, furthermore, the absence of a relationship in the offloading group may have been related to a ceiling effect due to better performance in this condition.

In a final set of analyses, the correlations between age and the other experimental tasks were calculated (collapsed across Experiments 2a and 2b, seeing as these tasks were identical in the two experiments). This showed that age was positively correlated with lexical decision accuracy (*r* = .26, *p* < 10^−15^), positively correlated with event-based PM (*r* = .30, *p* < 10^–21^), and negatively correlated with time-based PM (*r* = −.23, *p* < 10^−12^). Thus, older adults generally outperformed younger participants (possibly due to motivational factors, greater conscientiousness, etc.), but an age-related deficit in time-based PM was nevertheless revealed, consistent with previous results suggesting that age effects may be particularly pronounced in such tasks (e.g., Park, Hertzog, Kidder, Morrell, & Mayhorn, 1997).

In sum, the results were consistent with the hypothesis that older participants might be more likely to set external reminders to help remember delayed intentions. However, this result should be interpreted with caution, especially insofar as it relates the possibility of intention offloading as a compensatory strategy in older adults. First, it is not clear how well older and younger participants from Amazon Mechanical Turk are matched, seeing as there was little demographic information available about the participants. Second, seeing as older participants generally performed better on the experimental tasks, this finding might simply reflect greater motivation to perform the task well in older adults rather than any compensatory effect (indeed, there was no evidence that older adults had anything to compensate for in intention-offloading/nonoffloading task). When age was included as an additional independent variable/covariate in the analyses investigating the relationship between naturalistic PM and the experimental tasks, all results were similar.

## GENERAL DISCUSSION

This study investigated the relatively unexplored topic of intention offloading: acting on one's environment to create perceptual triggers for delayed intentions. Results of Experiment 1 showed that participants offload intentions adaptively, on the basis of the cognitive load of the task. Furthermore, Experiment 2 showed that the intention-offloading task and its matched nonoffloading control task were the best predictors of participants' likelihood of fulfilling a delayed intention integrated into their daily activities over a one-week period, indicating that the experimental paradigm had significant, albeit weak, external validity. Participants were more likely to offload intentions if (a) they had a greater memory load; (b) they encountered interruptions during the retention interval; and (c) they were older.

Experimental paradigms investigating participants' ability to fulfil delayed intentions typically do not permit intention offloading. Yet intention offloading was functionally related to task performance in the present studies—it boosted target accuracy. This suggests that typical experimental paradigms may miss additional variance contributing to performance when intention offloading is permitted. Understanding the metacognitive mechanisms contributing to intention offloading and other strategies may therefore provide additional understanding of the factors leading to the fulfilment of delayed intentions, beyond those measured by the performance of unaided tasks. Consistent with this suggestion, after matching the intention-offloading and nonoffloading task versions for mean target accuracy, only the intention-offloading task predicted performance of a naturalistic PM task. This suggests that allowing participants to set reminders increases the external validity of the task. However, seeing as the direct comparison between the two conditions did not show a significant difference, it is not possible to draw strong conclusions from this result.

Investigating intention-offloading strategies is potentially of practical significance. For example, the use of such strategies is presumably more amenable to behaviour-change interventions than participants' underlying unaided ability. With the development and widespread use of technological innovations such as smartphone reminder applications, delayed intentions can reliably be triggered in many situations (e.g., those involving time- or location-based cueing), if appropriate steps are taken in advance. Knowledge of the metacognitive and situational (e.g., ergonomic) factors that contribute to such steps being taken is therefore potentially important for improving the fulfilment of delayed intentions in everyday life (see Rummel & Meiser, 2013, for further discussion of the influence of metacognition on behaviour in a PM setting). Figure 3 suggested that few participants changed their offloading strategy from trial to trial. It is therefore an interesting question what factors might underlie individual differences in propensity to set reminders, even when the task is held constant. This is likely to involve a complex interplay of metacognitive and motivational factors (see Gilbert, 2014, for evidence that subjective confidence and objective ability independently predict the use of external reminders). It might be argued that once a reminder has been set, PM—at least as defined by some researchers—is no longer required, because the intended action can be directly cued. According to this view, the term “prospective memory” should be reserved for those situations that do not include the use of external artefacts. There is not necessarily any objective criterion by which to judge this terminological question. However, whatever one's view, reaching a broad understanding of the mechanisms that contribute to the fulfilment of delayed intentions is of practical significance, whether or not those mechanisms fall under a narrow definition of PM.

Intention offloading is not the only strategy that can be used to support the fulfilment of delayed intentions. It is therefore an interesting question as to what extent the metacognitive factors that lead to the triggering of different strategies are similar or distinct. For example, another common strategy is the use of “implementation intentions” (Gilbert, Gollwitzer, Cohen, Burgess, & Oettingen, 2009; Gollwitzer, 1999), where an intended thought or action is mentally linked with a specific anticipated situational cue. Intention offloading might be seen as an extreme form of implementation intention, where not only is an intention linked to an anticipated cue, but the agent physically interacts with the world to create a perceptual cue with a preexisting link to the intended behaviour. Thus, both strategies involve a form of prospection or simulation to anticipate the likely circumstances that will provide an appropriate trigger for a delayed intention (e.g., while forming an intention to make a phone call at 11 am tomorrow, I might think of my computer screen, or physically attach a post-it note, because that is where I expect to be looking at the relevant time).

As well as anticipating the appropriate time and place for a cue to appear when we offload intentions, it is also necessary to decide what aspects of the intention need to be offloaded. It is useful here to distinguish between prospective and retrospective elements of delayed intentions (Cohen, West, & Craik, 2001). The prospective element denotes the requirement to remember that something needs to be done at a particular time and the ability to trigger that intention when necessary. The retrospective element denotes the requirement to remember the appropriate behaviour once the prospective element has been triggered. In everyday life, it is clearly possible to distinguish offloading of the prospective versus the retrospective component of an intention. For example, I might create a content-free reminder (e.g., tying a knot in my handkerchief), which reminds me that something needs to be done, but not what it is (see Fish et al., 2007, for further discussion of content-free cueing). Alternatively, I might write the details of an appointment on a piece of paper, which reminds me of where I need to go, but only after I have remembered that I need to go somewhere and consulted this record. In the present intention-offloading task, a circle placed in a particular location could arguably cue both the prospective content of an intention (e.g., that I need to do something special when I get to number 5 in the sequence) as well as the retrospective content (e.g., what I need to do is drag it to the left). An alternative interpretation might be that the prospective element simply represents that *something* needs to be done on this trial, and the retrospective content is that circle 5 needs to be dragged to the left, in which case only the retrospective content was offloaded. Either way, it is an interesting possibility that intention offloading may depend on distinct mechanisms when supporting the prospective content of an intention, the retrospective content, or both. An additional consideration is that participants were explicitly provided with the intention offloading strategy in the present studies. However, the metacognitive mechanisms involved in generating a novel strategy may well differ from those investigated here, where participants implement a known strategy. Finally, it is of course possible that the metacognitive factors that relate to intention offloading over a short timescale, as in the present intention offloading task, may differ from those responsible for intention offloading over longer periods of minutes, days, or weeks.

In the data presented in this article, it was not possible to distinguish whether a failure to respond correctly to a target was caused by participants failing to remember their intention, failing to encode the intention to begin with, or incorrectly encoding the target identity or required response. These possibilities are difficult to distinguish behaviourally, but may be more amenable to a functional neuroimaging approach (for an adaptation of the present paradigm to an functional magnetic resonance imaging, fMRI, setting see Landsiedel and Gilbert, 2014). On a neurophysiological level, performance of unaided tasks requiring the fulfilment of delayed intentions has been linked to signal change in rostral prefrontal cortex (Burgess, Quayle, & Frith, 2001; Gilbert, 2011; Okuda et al., 1998), and patients with damage to this region show disorganization in everyday life (Burgess, 2000; Uretzky & Gilboa, 2010). This suggests that a core process involved in executing delayed intentions is supported by rostral prefrontal cortex (PFC). However, this region has also been implicated in generating metacognitive awareness of our own mental states (Baird, Smallwood, Gorgolewski, & Margulies, 2013; Fleming & Dolan, 2012; Fleming, Huijgen, & Dolan, 2012; Fleming, Weil, Nagy, Dolan, & Rees, 2010; McCurdy et al., 2013). This points to a potential additional way in which rostral PFC might contribute to everyday behavioural organization: by proactively triggering externalizing strategies (and indeed other strategies) in situations where unaided abilities are insufficient, with the consequence that subsequent demands on its role in fulfilling delayed intentions will be reduced. Understanding the ways in which such strategies are triggered, how they vary across individuals—in health and disease and across the lifespan—and how they influence subsequent behaviour can broaden our understanding of behavioural organization in everyday life.

## SUPPLEMENTAL MATERIAL

Supplemental material is available via the “Supplemental” tab on the article's online page (http://dx.doi.org/10.1080/17470218.2014.972963.2014).

## References

[CIT0001] Allen D. (2002). *Getting things done: How to achieve stress-free
productivity*.

[CIT0002] Altmann E. M., Trafton J. G. (2002). Memory for goals: An activation-based model. *Cognitive Science*.

[CIT0003] Altmann E. M., Trafton J. G., Hambrick D. Z. (2013). Momentary interruptions can derail the train of
thought. *Journal of Experimental Psychology: General*.

[CIT0004] Baird B., Smallwood J., Gorgolewski K. J., Margulies D. S. (2013). Medial and lateral networks in anterior prefrontal cortex support
metacognitive ability for memory and perception. *Journal of Neuroscience*.

[CIT0005] Bhandari A., Duncan J. (2014). Goal neglect and knowledge chunking in the construction of novel
behaviour. *Cognition*.

[CIT0006] Brandimonte M. A., Einstein G. O., McDaniel M. A. (1996). *Prospective memory: Theory and applications*.

[CIT0007] Burgess P. W. (2000). Strategy application disorder: The role of the frontal lobes in
human multitasking. *Psychological Research*.

[CIT0008] Burgess P. W., Quayle A., Frith C. D. (2001). Brain regions involved in prospective memory as determined by
positron emission tomography. *Neuropsychologia*.

[CIT0009] Burgess P. W., Veitch E., de Lacy Costello A., Shallice T. (2000). The cognitive and neuroanatomical correlates of
multitasking. *Neuropsychologia*.

[CIT0010] Clark A. (1997). *Being there: Putting brain, body, and world together again*.

[CIT0011] Clark A. (2010). *Supersizing the mind*.

[CIT0012] Clark A., Chalmers D. J. (2006). The extended mind. *Analysis*.

[CIT0013] Cohen A., West R., Craik F. I. M. (2001). Modulation of the prospective and retrospective components of
memory for intentions in younger and older adults. *Aging, Neuropsychology, and Cognition (Neuropsychology, Development
and Cognition: Section B)*.

[CIT0014] Craik F. I. M., Kerr S. A., Einstein G. O., McDaniel M. A. (1996). Commentary: Prospective memory, aging, and lapses of
intention. *Prospective memory: Theory and applications*.

[CIT0015] Crump M. J. C., McDonnell J. V., Gureckis T. M. (2013). Evaluating Amazon's mechanical turk as a tool for experimental
behavioral research. *PLoS ONE*.

[CIT0016] Duncan J. (2010). *How Intelligence Happens*.

[CIT0017] Duncan J., Emslie H., Williams P., Johnson R., Freer C. (1996). Intelligence and the frontal lobe: The organization of
goal-directed behavior. *Cognitive Psychology*.

[CIT0018] Duncan J., Parr A., Woolgar A., Thompson R., Bright P., Cox S., Nimmo-Smith I. (2008). Goal neglect and Spearman's g: Competing parts of a complex
task. *Journal of Experimental Psychology: General*.

[CIT0019] Einstein G. O., McDaniel M. A. (1990). Normal aging and prospective memory. *Journal of Experimental Psychology: Learning, Memory, and
Cognition*.

[CIT0020] Ellis J. A., Cohen G., Cohen G., Conway M. A. (2008). Memory for intentions, actions and plans. *Memory in the real world*.

[CIT0021] Field A. (2000). *Discovering statistics using SPSS for windows: Advanced techniques
for beginners (introducing statistical methods series)*.

[CIT0022] Fish J., Evans J. J., Nimmo M., Martin E., Kersel D., Bateman A., Manly T. (2007). Rehabilitation of executive dysfunction following brain injury:
“Content-free” cueing improves everyday prospective memory
performance. *Neuropsychologia*.

[CIT0023] Fish J., Wilson B. A., Manly T. (2010). The assessment and rehabilitation of prospective memory problems
in people with neurological disorders: A review. *Neuropsychological Rehabilitation*.

[CIT0024] Fleming S. M., Dolan R. J. (2012). The neural basis of metacognitive ability. *Philosophical Transactions of the Royal Society of London. Series B,
Biological Sciences*.

[CIT0025] Fleming S. M., Huijgen J., Dolan R. J. (2012). Prefrontal contributions to metacognition in perceptual decision
making. *Journal of Neuroscience*.

[CIT0026] Fleming S. M., Weil R. S., Nagy Z., Dolan R. J., Rees G. (2010). Relating introspective accuracy to individual differences in
brain structure. *Science*.

[CIT0027] Gilbert S. J. (2014).

[CIT0028] Gilbert S. J. (2011). Decoding the content of delayed intentions. *Journal of Neuroscience*.

[CIT0029] Gilbert S. J., Gollwitzer P. M., Cohen A.-L., Burgess P. W., Oettingen G. (2009). Separable brain systems supporting cued versus self-initiated
realization of delayed intentions. *Journal of Experimental Psychology: Learning, Memory, and
Cognition*.

[CIT0030] Gilbert S. J., Hadjipavlou N., Raoelison M. (2013). Automaticity and control in prospective memory: A computational
model. *PLoS ONE*.

[CIT0031] Gollwitzer P. M. (1999). Implementation intentions: Strong effects of simple
plans. *American Psychologist*.

[CIT0032] Graf P., Uttl B. (2001). Prospective memory: A new focus for research. *Consciousness and Cognition*.

[CIT0032a] Grömping, U. (2006). Relative importance for linear regression in R: The package relaimpo. *Journal of Statistical Software*, *17*, 1-27.

[CIT0033] Grundgeiger T., Sanderson P., MacDougall H. G., Venkatesh B. (2010). Interruption management in the intensive care unit: Predicting
resumption times and assessing distributed support. *Journal of Experimental Psychology: Applied*.

[CIT0034] Guynn M. J., McDaniel M. A., Einstein G. O. (1998). Prospective memory: When reminders fail. *Memory & Cognition*.

[CIT0035] Hall L., Johansson P., de Léon D., Clark A., Kiverstein J., Vierkant T. (2013). Recomposing the will: Distributed motivation and computer
mediated extrospection. *Decomposing the will*.

[CIT0036] Harris J. E. (1980). Memory aids people use: Two interview studies. *Memory & Cognition*.

[CIT0037] Henry J. D., Rendell P. G., Phillips L. H., Dunlop L., Kliegel M. (2012). Prospective memory reminders: A laboratory investigation of
initiation source and age effects. *The Quarterly Journal of Experimental Psychology*.

[CIT0038] Heylighen F., Vidal C. (2008). Getting things done: The science behind stress-free
productivity. *Long Range Planning*.

[CIT0039] Hutchins E. (1995). *Cognition in the wild*.

[CIT0040] Kane M. J., Conway A. R. a, Miura T. K., Colflesh G. J. H. (2007). Working memory, attention control, and the N-back task: A
question of construct validity. *Journal of Experimental Psychology: Learning, Memory, and
Cognition*.

[CIT0041] Kirsh D. (1995). The intelligent use of space. *Artificial Intelligence*.

[CIT0042] Kirsh D. (1996). Adapting the environment instead of oneself. *Adaptive Behavior*.

[CIT0043] Kliegel M., McDaniel M. A., Einstein G. O. (2008). *Prospective memory: Cognitive, neuroscience, developmental, and
applied perspectives*.

[CIT0044] Koechlin E., Basso G., Pietrini P., Panzer S., Grafman J. (1999). The role of the anterior prefrontal cortex in human
cognition. *Nature*.

[CIT0045] Landsiedel J., Gilbert S. J. (2014).

[CIT0046] Lindenberger U., Mayr U. (2014). Cognitive aging: Is there a dark side to environmental
support?. *Trends in Cognitive Sciences*.

[CIT0047] Loft S., Smith R. E., Bhaskara A. (2011). Prospective memory in an air traffic control simulation: External
aids that signal when to act. *Journal of Experimental Psychology: Applied*.

[CIT0048] Logie R. H., Maylor E. A. (2009). An Internet study of prospective memory across
adulthood. *Psychology and Aging*.

[CIT0049] Loukopolous L. D., Dismukes R. K., Barshi I. (2009). *The multitasking myth*.

[CIT0050] Maylor E. A. (1990). Age and prospective memory. *The Quarterly Journal of Experimental Psychology A*.

[CIT0051] Maylor E. A., Kliegel M. A., McDaniel M. A., Einstein G. O. (2008). Commentary: Prospective memoroy through the ages. *Prospective memory: Cognitive, neuroscience, developmental, and
applied perspectives*..

[CIT0052] McCurdy L. Y., Maniscalco B., Metcalfe J., Liu K. Y., de Lange F. P., Lau H. (2013). Anatomical coupling between distinct metacognitive systems for
memory and visual perception. *Journal of Neuroscience*.

[CIT0053] McDaniel M. A., Einstein G. O. (2000). Strategic and automatic processes in prospective memory
retrieval: A multiprocess framework. *Applied Cognitive Psychology*.

[CIT0054] Menary R. (2010). *The extended mind*.

[CIT0055] Okuda J., Fujii T., Yamadori A, Kawashima R., Tsukiura T., Fukatsu R., Fukuda H. (1998). Participation of the prefrontal cortices in prospective memory:
Evidence from a PET study in humans. *Neuroscience Letters*.

[CIT0056] Park D. C., Hertzog C., Kidder D. P., Morrell R. W., Mayhorn C. B. (1997). Effect of age on event-based and time-based prospective
memory. *Psychology and Aging*.

[CIT0057] Phillips L. H., Henry J. D., Martin M., Kliegel M., McDaniel M. A., Einstein G. O. (2008). Adult aging and prospective memory: The importance of ecological
validity. *Prospective memory: Cognitive, neuroscience, developmental, and
applied perspectives*..

[CIT0058] Poldrack R. (2006). Can cognitive processes be inferred from neuroimaging
data?. *Trends in Cognitive Sciences*.

[CIT0059] Poldrack R. A. (2011). Inferring mental states from neuroimaging data: From reverse
inference to large-scale decoding. *Neuron*.

[CIT0060] Ratcliff R., Thapar A., McKoon G. (2010). Individual differences, aging, and IQ in two-choice
tasks. *Cognitive Psychology*.

[CIT0061] Reynolds J. R., West R., Braver T. (2009). Distinct neural circuits support transient and sustained
processes in prospective memory and working memory. *Cerebral Cortex*.

[CIT0062] Robertson I. H., Manly T., Andrade J., Baddeley B. T., Yiend J. (1997). “Oops!”: Performance correlates of everyday
attentional failures in traumatic brain injured and normal
subjects. *Neuropsychologia*.

[CIT0063] Roca M., Torralva T., Gleichgerrcht E., Woolgar A., Thompson R., Duncan J., Manes F. (2011). The role of Area 10 (BA10) in human multitasking and in social
cognition: A lesion study. *Neuropsychologia*.

[CIT0064] Rummel J., Meiser T. (2013). The role of metacognition in prospective memory: Anticipated task
demands influence attention allocation strategies. *Consciousness and Cognition*.

[CIT0065] Scullin M. K., McDaniel M. A., Einstein G. O. (2010). Control of cost in prospective memory: Evidence for spontaneous
retrieval processes. *Journal of Experimental Psychology: Learning, Memory, and
Cognition*.

[CIT0066] Scullin M. K., McDaniel M. A., Shelton J. T. (2013). The dynamic multiprocess framework: Evidence from prospective
memory with contextual variability. *Cognitive Psychology*.

[CIT0067] Shallice T., Cooper R. (2011). *The organisation of mind*.

[CIT0068] Sheeran P. (2002). Intention—Behavior relations: A conceptual and empirical
review. *European Review of Social Psychology*.

[CIT0069] Spearman C. (1904). “General intelligence,” objectively determined and
measured. *The American Journal of Psychology*.

[CIT0070] Svoboda E., Rowe G., Murphy K. (2012). From science to smartphones: Boosting memory function one press
at a time. *Journal of Current Clinical Care*.

[CIT0071] Thöne-Otto A. I. T., Walther K., Kliegel M., McDaniel M. A., Einstein G. O. (2008). Assessment and treatment of prospective memory disorders in
clinical practice. *Prospective memory: Cognitive, neuroscience, developmental, and
applied perspectives*..

[CIT0072] Uretzky S., Gilboa A. (2010). Knowing your lines but missing your cue: Rostral prefrontal
lesions impair prospective memory cue detection, but not action-intention
superiority. *Journal of Cognitive Neuroscience*.

[CIT0073] Uttl B. (2008). Transparent meta-analysis of prospective memory and
aging. *PLoS ONE*.

[CIT0074] Uttl B., Kibreab M. (2011). Self-report measures of prospective memory are reliable but not
valid. *Canadian Journal of Experimental Psychology*.

[CIT0075] Vortac O. U., Edwards M. B., Manning C. A. (1995). Functions of external cues in prospective memory. *Memory*.

[CIT0076] Wilson B. A., Emslie H. C., Quirk K., Evans J. J. (2001). Reducing everyday memory and planning problems by means of a
paging system: A randomised control crossover study. *Journal of Neurology, Neurosurgery, & Psychiatry*.

[CIT0077] Wright P., Fields R., Harrison M. (2000). Analyzing human-computer interaction as distributed cognition:
The resources model. *Human-Computer Interaction*.

